# Extensive Tympanic Membrane Cholesteatoma with Marginal Perforation: An Unusual Case

**DOI:** 10.1155/2013/865043

**Published:** 2013-07-14

**Authors:** Erdal Sakalli, Deniz Kaya, Cengiz Celikyurt, Selcuk Cem Erdurak

**Affiliations:** ^1^Safa Private Hospital, Department of Otorhinolaryngology, Istanbul, Turkey; ^2^Fevzi Çakmak Mahallesi Şişecam Bloklari, Emek Apartment D:8, Bağcilar, Istanbul, Turkey; ^3^Hisar Intercontinental Private Hospital, Department of Otorhinolaryngology, Istanbul, Turkey

## Abstract

The migration of squamous epithelium of external ear through a tympanic membrane perforation into the middle ear forms a cholesteatoma. But it is extremely a rare condition to observe extensive cholesteatoma on the medial surface of tympanic membrane with perforation. This condition is termed tympanic membrane cholesteatoma (TMC). We herein present an exceptional case of extensive TMC with marginal perforation.

## 1. Introduction

Cholesteatoma is simply the presence of squamous epithelium in an aberrant location. It can involve any area of the middle ear, hypotympanum, protympanum, epitympanum, and mastoid. A minimal degree of cholesteatoma through a tympanic membrane (TM) perforation can sometimes be observed on the medial surface of the tympanic membrane in tympanic surgery. But it is unusual to find extensive cholesteatoma on the medial surface of a perforated TM [1].

## 2. Case Report

**Figure 1:**
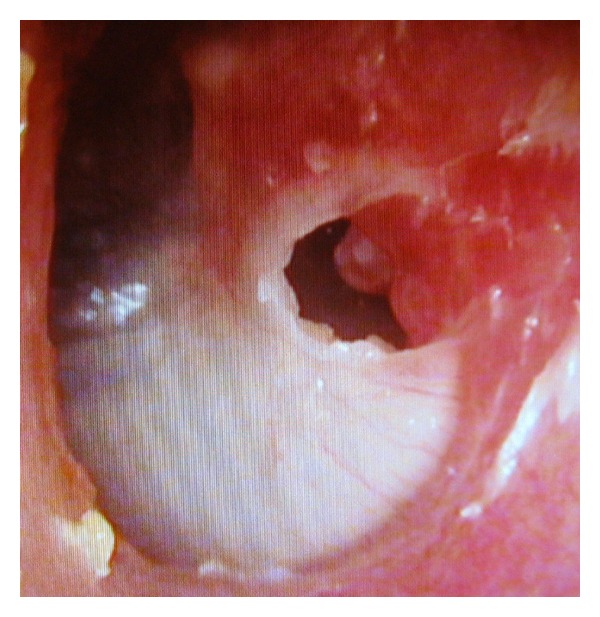
Otoendoscopic view of the left ear with a white lesion localized in the posterosuperior, posteroinferior, and anteroinferior quadrants of the tympanic membrane with marginal perforation.

**Figure 2:**
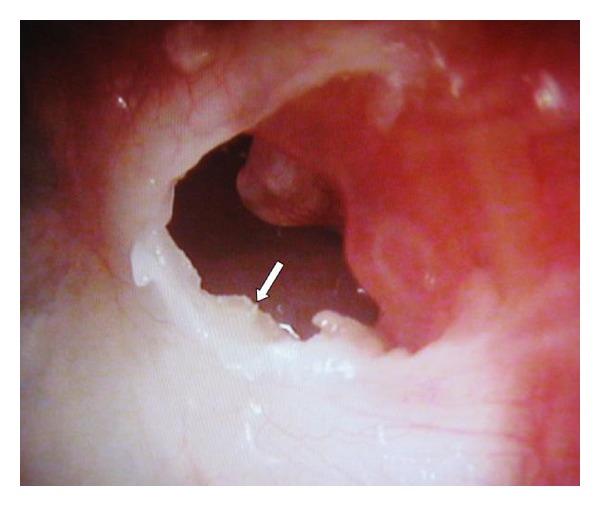
Otoendoscopic view of the marginal perforation of the left ear. White keratin debris appears on the medial surface of the tympanic membrane from the perforation (arrow: white keratin debris).

**Figure 3:**
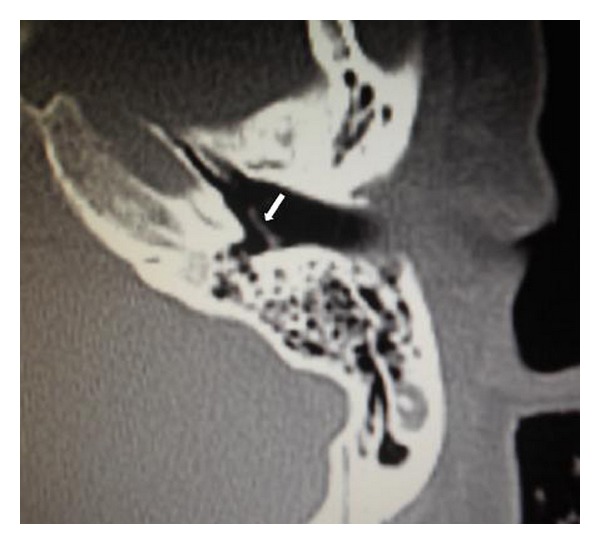
Axial computed tomography scan of the left temporal bone showed thickening of the tympanic membrane with a normal middle ear and mastoid cavity (arrow: tympanic membrane).

A 47-year-old female patient presented with recurrent purulent discharge from her left ear for approximately 10 years. She visited primary care physicians several times and was prescribed topical drugs that temporarily alleviated the otorrhea. She had no history of trauma or ear surgery on her left ear. Audiometry demonstrated asymptomatic 30 dB conductive hearing loss in the left ear. Otomicroscopy revealed a white TM in the normal position with a true marginal perforation in the posterosuperior quadrant (Figure 1). The posterosuperior, posteroinferior, and anteroinferior quadrants of the TM appeared white, opaque, and thick. The anterosuperior quadrant of the TM was grayish and semitransparent. White keratin debris was coming out of the perforation from the medial surface, and there was some granulation tissue on the retrotympanum at the posterior edge of the perforation (Figure 2). The mucosa of medial tympanic wall looked normal. The CT scan of the temporal bone showed thickening of the TM (Figure 3). 

Tympanoplasty using a retroauricular approach was performed. When the tympanomeatal flap was elevated, squamous epithelium with a thin layer of white keratin was found to cover the medial surface of the TM. Along the edge of the perforation, squamous epithelium on the lateral surface of the TM was in continuity with that on the medial surface of the TM. The white color of the cholesteatoma helped to define the involved parts of the middle ear mucosa. The white parts of the TM, the adjacent middle ear mucosa, and granulation tissue were totally removed. Underlay tympanoplasty was performed using a temporal facial graft. Histopathologic examination confirmed the diagnosis of tympanic membrane cholesteatoma (TMC). 

## 3. Discussion 

Cholesteatomas are classified as congenital or acquired. The literature contains many reports of congenital cholesteatoma [2]. Congenital cholesteatoma develops within an absolutely normal TM between the lateral and medial surfaces of the TM without any prior history of otologic surgery, trauma, otorrhea, or TM perforation [3]. In our case, we observed a marginal perforation in the posterosuperior quadrant with a history of otorrhea. These findings were consistent with an acquired, not congenital, TMC.

Although the pathogenesis of acquired cholesteatoma continues to be controversial, some theories regarding its pathogenesis have been suggested. In 1899, Bezold [4] and Haberman [5] first proposed the concept of migration of squamous epithelium from the external auditory canal and/or the TM through a perforation of the TM into the middle ear to form a cholesteatoma. Karmody and Northrop presented histologic findings supporting the migration theory that cholesteatomas are formed by medial migration of the stimulated squamous epithelium of the TM [6]. 

TMC was first described in 1984 by Graham et al. [1]. They noted four cases of TMC with central TM perforations. In those cases, the epithelium did not develop in a saccular manner but simply lined the undersurface of the TM, actively desquamating epithelial debris onto the promontory. They characterized the TMC as acquired and originating from a central perforation, apparently confined to the undersurface of the pars tensa, without encapsulation or sacculation of the squamous epithelium, and with active desquamation of debris into the otherwise normal middle ear. The majority of our findings are in accordance with those of Graham et al. [1]. In our case, there was a marginal perforation and the squamous epithelium of the lateral layer of the TM appeared to migrate toward the medial surface of the TM. 

TMC can occur as a complication of any otologic surgery, especially tympanoplasty when the graft is placed lateral to the remaining TM, which can retract or invaginate, producing self-enclosed keratin debris. However, an extensive cholesteatoma on the medial surface of the TM without any history of ear surgery is an unexpected condition [7]. TMC occurs without ear surgery and forms a white lesion on a perforated TM. White squamous epithelium and desquamations of keratin debris coming out of the perforation from the medial surface of the TM are characteristics of TMC [1]. 

 Although TMC is very rare, it should be considered as a differential diagnosis of white TM lesions. Careful otomicroscopic examination and aspiration are necessary for white TM lesions with perforation. Treatment comprises accurate diagnosis of TMC, total removal of the white parts of the TM, and closure of the resultant large TM defect with graft material. Failure to diagnose TMC early can lead to progressive destruction, as in any type of cholesteatoma.
